# Recent Green and Sustainable Pd‐Catalyzed Aminations

**DOI:** 10.1002/cssc.202500184

**Published:** 2025-05-21

**Authors:** John Michael Saunders, Kylee B. Dismuke Rodriguez, Robert M. Lammert, Jordan R. Yirak, Karthik S. Iyer, Madison J. Wong, Bruce H. Lipshutz

**Affiliations:** ^1^ Department of Chemistry & Biochemistry University of California Santa Barbara CA 93106 USA

**Keywords:** aminations, chemistry in water, flow, micellar catalysis, recyclability, surfactants, sustainability

## Abstract

Pd‐catalyzed aminations, a powerful and commonly employed method of C—N bond construction, often rely on unsustainable technologies that utilize egregious organic solvents, high temperatures, long reaction times, and high catalyst loadings, especially of palladium. Only recently there has been a shift toward far greener protocols based on recyclable aqueous media as well as nontraditional organic solvents. In addition, alternatives to batch methods for preparing the same amines have appeared, such as continuous flow and mechanochemistry, which also offer safe and sustainable means of chemical synthesis associated with targets in the fine chemicals industry.

## Graphical abstract

Herein, several sustainable methods toward Pd‐catalyzed aminations of aryl/heteroaryl (pseudo)halides with alkyl and (hetero)aryl amines are discussed. The use of bio‐derived solvents or recyclable aqueous micellar media allows for reduced Pd loadings without using egregious organic solvents. Similarly, under continuous flow or mechanochemical means, increased reaction rates allow for faster aminations, using low levels of a Pd catalyst.

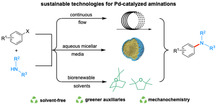

## 1 Introduction

Sustainability is no longer just a word that occasionally makes it into someone's prose. It is fair to say that today, it is a buzzword seen routinely, especially in the chemistry community. It entails achieving a balance between meeting societal needs while preserving the environment and ensuring the responsible management of natural resources. The sustainable use and manufacturing of agro‐ and fine chemicals began in the wake of Rachel Carson's *Silent Spring* ^[^ ^1^ ^]^ in 1962. Her exposé on the misuse of chemicals, such as dichlorodiphenyltrichloroethane (DDT), caused a turning point associated with the use of agrochemicals. However, with the world's ever‐growing population, there is an inherent need to produce not only agrochemicals but also the drugs or active pharmaceutical ingredients (APIs) that will keep people alive. Thus, in 1998, Anastas and Warner set forth the *12 Principles of Green Chemistry* ^[^ ^2^ ^]^ as a guide toward sustainable chemical production. These principles focus on many key factors that, after a quarter of a century, have become more in focus every day; perhaps climate change has forced attention to what now seem like obvious concerns, such as preventing waste, high atom economy, reduced energy demands, and catalytic versus stoichiometric transformations, to name only a few. In brief, reducing the demand for both materials and energy required by every chemical reaction helps improve sustainability.

From a sustainability perspective, multiple factors come into play. Variables include reaction temperature and duration, both of which impact energy consumption and, usually, cost. Workup that may require additional reagents, choice of solvent, and strategies for waste management are also important parameters. Balancing these while realizing high product yields can be challenging. Tools such as Sheldon's E‐Factor^[^ ^3^, ^4^, ^5^, ^6^ ^]^ or the use of the industry‐favored metric process mass intensity (PMI)^[^ ^7^ ^]^ provide quantitative measures to assess the “greenness” of a process by considering most aspects of a process. Ultimately, a life cycle assessment (LCA) is rapidly gaining prominence as a preferred method of analysis. This is mainly used in industrial circles for evaluating the overall sustainability of chemical reactions as revealed via their environmental footprints.^[^ ^8^ ^]^ This is often done by measuring the total contributions, starting from the production of raw materials to the market product (cradle‐to‐gate), or in a more holistic manner, to the waste products and their methods of treatment (cradle‐to‐grave).

Since the usage of organic solvents represents a large portion of waste generated in the synthesis of APIs,^[^ ^9^ ^]^ special attention continues to be directed toward safer, more sustainable, and renewable solvents used in responsible ways. Often, using nature's chosen solvent (i.e., water) reduces waste generation, as well as leads to superior alternative technologies relative to those that traditionally rely on organic solvents. Ideally, there would be no need for the use of any solvent (i.e., neat reactions). Likewise, in terms of reagents, their use under sustainable conditions is critical, with catalysis an important means of accomplishing this goal. When possible, chemists should avoid selecting endangered transition metals (such as Pd, Ni, and Cu),^[^ ^10^ ^]^ although it is often unavoidable that Earth‐abundant metal usage must be considered hand in hand with their chemistry being run in water. Fortunately, uses of transition metals that facilitate key C—C bond constructions via cross‐coupling reactions, such as Suzuki–Miyaura,^[^ ^11^, ^12^ ^]^ Sonogashira,^[^ ^13^ ^]^ and Negishi^[^ ^14^, ^15^ ^]^ couplings, no longer require organic solvents, avoid unsustainable levels of precious metal catalyst, and offer a workup protocol that eliminates additional use of water. However, there are several other types of important bonds that are also catalyzed by palladium, and these must also be addressed in terms of sustainability.

**Scheme 1:**
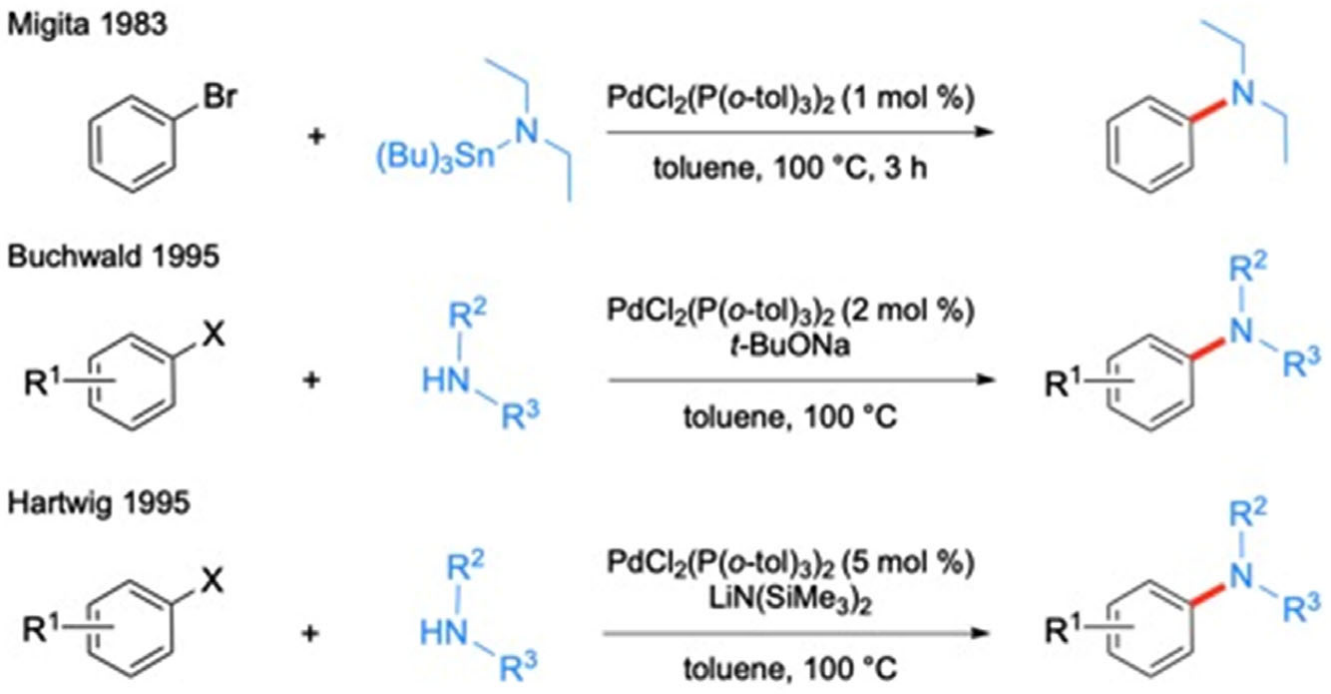
General schemes for the coupling of amines and aminostannanes with aryl halides using catalytic Pd.

Due to the commonality of nitrogen being found within pharmaceuticals, agrochemicals, and other fine chemicals, attention has rightly been directed toward their synthesis, in particular from the perspective of sustainability.^[^ ^16^ ^]^ Traditionally, the construction of C(sp^2^)—N bonds is facilitated by nucleophilic aromatic substitution^[^ ^17^ ^]^ or Cu‐catalyzed Ullman^[^ ^18^, ^19^ ^]^ couplings. While strides have been made that enable such key bond formations, each remains a considerable distance from being called “sustainable,” given that they typically use relatively high loadings of metals, oftentimes require high temperatures, and are typically carried out in environmentally egregious organic solvents. In 1983, long before sustainability was an issue, Migita et al. began working on the coupling of aminostannanes with aryl bromides resulting in aryl amines, albeit with a limited substrate scope.^[^ ^20^ ^]^ Soon thereafter, both Buchwald and Hartwig independently published their tin‐free synthesis of aryl amines via C—N bond construction, now known as Buchwald–Hartwig aminations (**Scheme** **1**).^[^ ^21^, ^22^ ^]^ Their systems obviated aminostannanes by incorporating a strong base, although the loading of palladium was doubled. This increase in the amount of catalyst was perfectly reasonable for the times, although today, reliance on 1–5 mol % of a precious metal catalyst is not only costly but surely unsustainable.

Since these early reports, aminations have become an important standard in the cross‐coupling between (hetero)aryl (pseudo)halides and amines.^[^ ^16^, ^23^, ^24^, ^25^, ^26^ ^]^ Unfortunately, and notwithstanding the passing of 30 years since the appearance of these first reports, progress has primarily focused on ligand design^[^ ^27^, ^28^, ^29^, ^30^, ^31^ ^]^ and thereby, to some degree, substrate scope. The uses of organic solvents, such as 1,4‐dioxane, THF, and DME with elevated temperatures and catalyst loadings over time (usually 12–24 h) are standard reaction conditions for Pd‐catalyzed aminations.^[^ ^26^, ^32^ ^]^ However, recent efforts toward more sustainable methods of aminations have appeared, where, rather than focusing on ligand design, there is an overall goal of achieving sustainable aminations. Thus, attention is now aimed at reducing palladium loadings, recycling the palladium catalyst, investing less energy through milder reaction conditions, and using less hazardous solvents to address safety and waste concerns. Hence, this review is directed toward Pd‐catalyzed aminations developed over roughly the past decade, showcasing significant progress en route to sustainable C—N bond formation, using 1) chemistry in water; 2) sustainable replacements for organic solvents; and 3) mechanochemical and continuous flow methods. Lastly, representative applications of sustainable protocols to targets in the pharmaceutical and fine chemicals industry are discussed.

## 2 Sustainable Aminations Using Chemistry in Water

**Figure 1:**
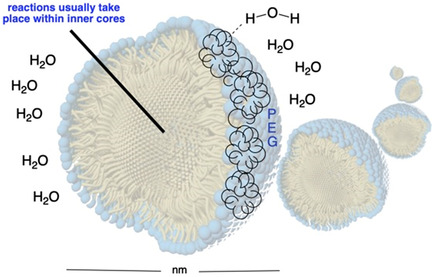
Self‐assembled nanomicelles in water.

While metrics such as E‐Factor, PMI, and LCA are valuable, they often fail to account for the environmental hazards associated with specific reagents or the ultimate fate of waste products and byproducts resulting from the transformation. This creates a significant opportunity for the development of recyclable reaction media that, even when disposed of, pose no harm to ecosystems and require minimal treatment prior to disposal. Important contributions in this area have been made by designing innovative surfactants that enable chemical transformations in water. These surfactants self‐assemble into micelles in aqueous media, allowing for the solubilization of organic molecules and facilitating reactions in an environmentally benign medium, water (**Figure** **1**). Within the micellar core, relative reactant concentrations are increased, enabling faster reaction rates, reduced temperatures, and lower catalyst loadings.^[^ ^33^ ^]^

**Figure 2:**
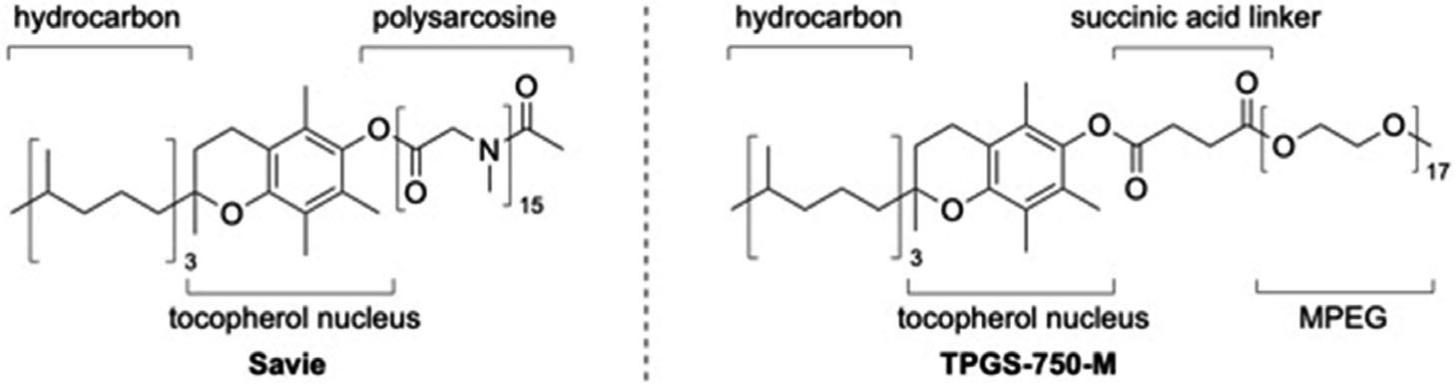
Structure of the surfactants Savie and TPGS‐750‐M.

A very recent contribution in the form of a new designer surfactant, Savie,^[^ ^34^ ^]^ represents a significant advancement in this field, affording experimental results that appear to be as good or better than those obtained in micelles derived from the original “workhorse” surfactant TPGS‐750‐M, first introduced back in 2011 (**Figure** **2**). One of Savie's major features, inspired by comments made by Novartis in Basel, includes a polysarcosine chain (with “DMF‐like” properties), which is well‐known to be biodegradable, hence simplifying the downstream processing of aqueous reaction mixtures. This amphiphile has been successfully employed in constructions of C—N bonds, leveraging both homogeneous and heterogeneous systems that achieve these transformations efficiently in water.^[^ ^35^, ^36^, ^37^, ^38^ ^]^

### 2.1 Heterogeneous Nanoparticle (NP) Catalysis for ppm Pd‐Catalyzed Aminations in Water

**Scheme 2:**
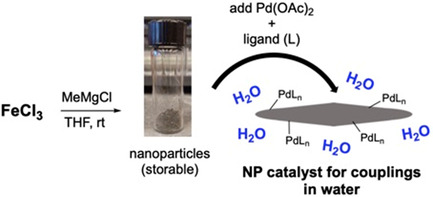
Preparation of Pd‐containing NPs for use in aminations.

Pd‐catalyzed C—N couplings have been carried out in aqueous solutions of Savie (2 wt %), the first study leveraging NPs prepared in a single step from FeCl_3_, assisted by the “nano‐to‐nano” effect.^[^ ^39^ ^]^ This phenomenon arises from the attraction between nanomicelles and metal NPs in water, since the Pd positioned on the NPs is “looking for electrons” to arrive at or, at least approach, the 18‐electron stability it seeks. Since the MPEG portion of TPGS‐750‐M has many oxygens, each bearing lone pairs of electrons, the micellar array “delivers” the substrate localized within to the catalyst (Pd‐containing NPs) for this reason, enabling various transformations to proceed under mild, environmentally friendly conditions with remarkably low precious metal loadings. Initially, these NPs were developed as catalysts for Negishi couplings^[^ ^40^ ^]^ pre‐ligated with specific ligands. Subsequently, a generalized preparation of NPs was introduced,^[^ ^41^ ^]^ where the metal and ligand are added to the NPs in water immediately before use, thereby guaranteeing catalyst activity (**Scheme** **2**).

**Scheme 3:**
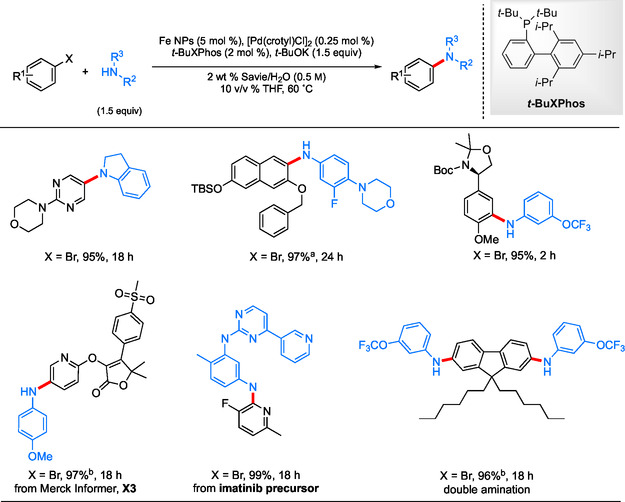
Selected scope for the Fe NP‐catalyzed aqueous aminations of aryl halides with anilines. a) Amine HCl was used; b) [Pd(crotyl)Cl]_2_ (0.175 mol % Pd).

Using the same NP technology but with a change of ligand to *t*‐BuXPhos, a general protocol was developed for heterogeneous catalysis facilitating ppm*‐*level Pd‐catalyzed C—N cross‐coupling reactions under aqueous micellar conditions.^[^ ^35^ ^]^ Again, and unlike most heterogeneous reactions that require elevated temperatures due to the need for increased rates of collision, these take place under mild reaction conditions enabled due to the nano‐to‐nano effect, leading to efficient C—N bond formation across a broad scope of (hetero)aryl halides and anilines. As an added benefit, both the aqueous medium and catalyst could be recycled. Notably, this technology has been successfully applied to the amination of several APIs, and their precursors, as well as substrates from the Merck Informer Library,^[^ ^42^, ^43^, ^44^ ^]^ all leading to the corresponding amines in excellent isolated yields (**Scheme** **3**). In some cases, a co‐solvent is used to assist with “softening” a lattice or even dissolution of highly crystalline substrates. This can dramatically enhance reaction rates if needed. The amount and nature of each must be determined experimentally, but such information can fortunately be obtained quickly (e.g., by thin layer chromotography analysis of small‐scale reactions run side‐by‐side, simply gauging the extent of conversion).

### 2.2 Pd‐Catalyzed Aminations with Aliphatic Amines in Water

**Scheme 4:**
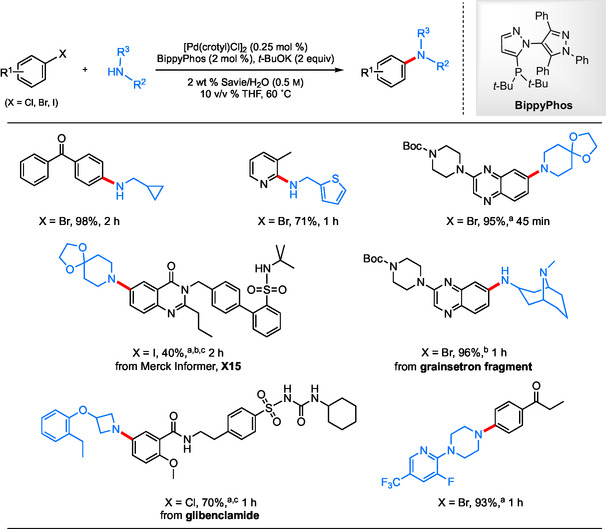
Selected scope for the aqueous aminations of aryl halides with alkyl amines using BippyPhos. a) Amine HCl was used; b) [Pd(crotyl)Cl]_2_ (0.375 mol % Pd).

Another recent methodology resulting in C—N bond formation in water utilizes a similar catalytic system; however, these same NPs were not required when aliphatic amines were used as coupling partners.^[^ ^35^ ^]^ This discovery led to the development of a new catalytic system employing the bipyrazole‐based ligand BippyPhos,^[^ ^45^ ^]^ which has demonstrated an exceptionally broad scope for Pd‐catalyzed C—N couplings.^[^ ^46^ ^]^ This work highlights the successful coupling of primary, secondary, and benzylic amines with a diverse range of functionalized substrates in good‐to‐excellent yields (**Scheme** **4**).

**Scheme 5:**
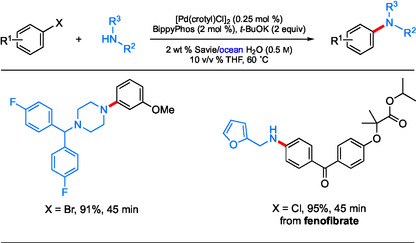
Scope of BippyPhos‐catalyzed aqueous aminations of aryl halides with alkyl amines using ocean water.

Notably, this study also includes two representative C—N couplings conducted in ocean water (**Scheme** **5**), showcasing this reaction medium serving as a viable and cost‐effective alternative. By simply filtering any undissolved solids and degassing under a stream of argon, the requisite 2 wt % surfactant in ocean water can be prepared and used in the same manner as with other surfactant solutions. No special procedure was needed beyond this initial treatment either up front or at the back end (i.e., purification).

**Scheme 6:**
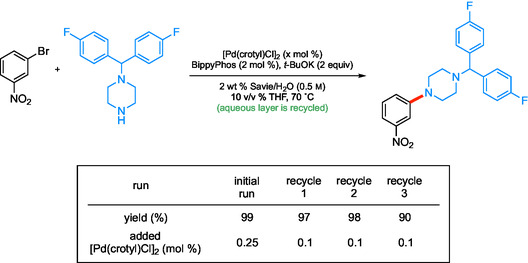
Recycling study for reuse of aqueous surfactant solution toward aliphatic aminations using reduced Pd each recycle.

A recycling study has also been conducted to highlight the advantages of using a recyclable aqueous medium. As expected, the E‐Factor and PMI after each coupling reaction pointed to the “greenness” of the newly developed process. Since either value is mainly based on the amount of solvent involved, the desired product was isolated via an in‐flask extraction using recyclable MTBE. Fresh catalyst, ligand, base, and starting materials were then added to the same aqueous medium for subsequent reactions (**Scheme** **6**). Based on this approach, the calculated E‐Factor was impressively low at 1.51 using recyclable MTBE, whereas this value increased to only 10.5 when MTBE was considered waste. Furthermore, this recycling strategy leads to the use of even lower amounts of Pd catalyst than required in the original coupling, as a portion of the original palladium likely remains active within the aqueous medium.

### 2.3 A New Pd Oxidative Addition Complex (OAC) for Aminations of Aryl Fluorosulfates

Another new approach to aminations was also described in 2024, focused on the use of aryl fluorosulfates as leaving groups in place of triflates and nonaflates (which are classified as polyfluorinated alkyl substances or PFAS).^[^ ^47^ ^]^ Although aryl fluorosulfates were first described in the 1970s,^[^ ^48^ ^]^ their resurgence is driven in part by the introduction of new and efficient methods for their preparation from phenolic starting materials.^[^ ^49^, ^50^, ^51^, ^52^, ^53^, ^54^ ^]^ These advancements have positioned aryl fluorosulfates as attractive, sustainable alternatives to traditional PFAS‐based pseudohalides.

**Scheme 7:**
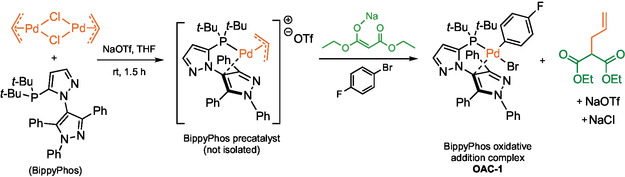
Glovebox‐free synthesis of **OAC‐1** containing the BippyPhos ligand.

Aminations of aryl/heteroaryl fluorosulfates in water rely on a Pd‐based catalytic system that involves a new OAC, “**OAC‐1.**”^[^ ^37^ ^]^ Through extensive ligand screening that involved evaluating over 30 candidates, BippyPhos exhibited the highest activity toward C—N bond construction using fluorosulfates. Based on a modified protocol developed by Pfizer,^[^ ^55^ ^]^ **OAC‐1** could be prepared in a one‐pot fashion without the use of a glovebox (**Scheme** **7**). Notably, this OAC is bench, air, and moisture stable and can be conveniently purified using silica gel.

**Scheme 8:**
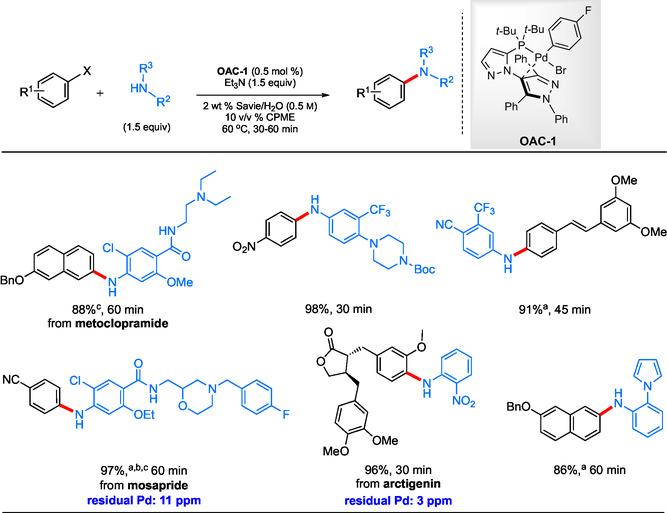
Selected scope for the **OAC‐1**‐catalyzed aqueous aminations of aryl fluorosulfates with anilines. a) **OAC‐1** (0.75 mol %); b) 80 °C; c) ArOSO_2_F(1.5 equiv), amine (1 equiv).

Based on early results,^[^ ^37^ ^]^ 0.5 mol % Pd in the form of **OAC‐1** resulted in efficient amination within minutes, significantly faster than the 2 h required using the Pd dimer and ligand separately. A broad range of couplings between aryl fluorosulfates and substituted anilines was found to be amenable (**Scheme** **8**). The protocol is particularly robust, accommodating functionalized substrates as well as couplings of APIs under mild and sustainable conditions.

### 2.4 An Application of Pd‐Catalyzed Aminations in Water: Erdafitinib

One example of C—N bond formation within the context of API synthesis calls attention to the anticancer agent erdafitinib.^[^ ^38^ ^]^ The original synthetic pathways developed by Astex in 2011 employed Pd‐catalyzed aminations using either DME or dioxane, both at elevated temperatures.^[^ ^56^ ^]^ From the green chemistry perspective, use of DME as a solvent is not encouraged, while dioxane is notoriously dangerous, not to mention its known adverse health effects.^[^ ^57^ ^]^ In both cases, the high temperatures and extended reaction times also pose significant sustainability challenges.

**Scheme 9:**
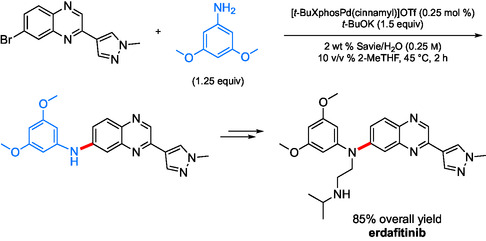
Amination used in the synthesis of the drug erdafitinib.

To overcome these issues, a novel three‐step, two‐pot route using water as the reaction medium was developed. It features an impressive 85% overall yield, including a total Pd loading of 0.75 mol %, a substantial improvement in both efficiency and sustainability (**Scheme** **9**).

Compared to the previously reported synthetic routes, the E‐Factor associated with the amination step (run in 1,4‐dioxane) was dramatically reduced from 128.5^[^ ^56^ ^]^ to 12.3.^[^ ^38^ ^]^ This underscores the advantages of employing a green reaction medium: lower Pd loadings, milder reaction conditions, and faster reaction rates, all while eliminating the safety hazards associated with aminations carried out in toxic organic solvents.^[^ ^57^ ^]^

### 2.5 Aminations in Aqueous Surfactant TPGS‐750‐M

**Scheme 10:**
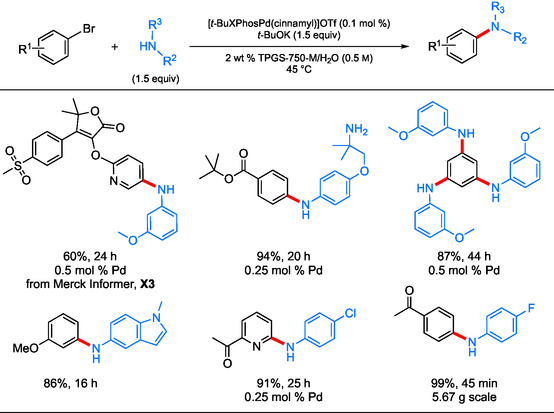
Selected cases of Pd‐catalyzed aminations of aryl halides with anilines using aqueous TPGS‐750‐M.

Although C—N couplings in Savie are likely to increase in prominence, the “workhorse” surfactant TPGS‐750‐M^[^ ^58^ ^]^ has been found to provide an aqueous medium for the intended catalysis that is quite enabling (see Figure 2). Its dissolution in water leads to a micellar medium that facilitates micellar catalysis, leading to targeted C—N bonds being formed under mild temperatures (45 °C) and with low Pd loadings (0.1–0.5 mol %; **Scheme** **10**).^[^ ^59^ ^]^ Aminated products include several late‐stage functionalizations, including substrates showing selectivity toward anilines over aliphatic amines, all in good‐to‐excellent yields. To showcase the reactivity of the initially introduced [*t*‐BuXPhosPd(cinnamyl)]OTf catalyst, a major contribution first introduced by Colacot,^[^ ^60^ ^]^ simultaneous double and even triple aminations could be conducted on single‐aryl halides. A gram‐scale reaction was also described to illustrate the potential for scale‐up, affording a 99% yield of the desired amine where, when run in toluene or dioxane, no reaction took place. Several additional direct comparisons to meaningful literature examples were also provided, illustrative of the improved yields obtained, as well as the green and sustainable conditions under which these aminations can now be run.

### 2.6 Mild Conditions for C—N Bond Formation Using Weak Bases in Water

**Scheme 11:**
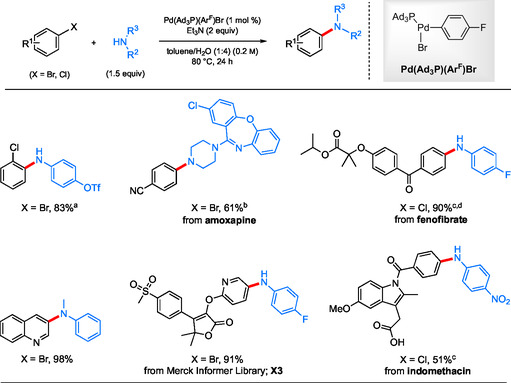
Selected examples of water‐assisted aminations using an OAC prepared from the commercially available Ad_3_P ligand. a) Neat water; b) 48 h; c) 100 °C; d) 36 h.

**Figure 3:**
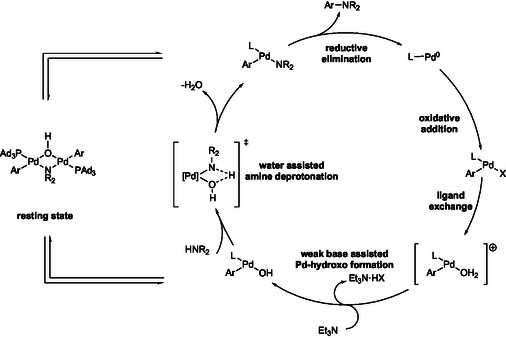
Proposed mechanistic pathway for the water‐assisted aminations using weak base.

Carrow et al. described an aqueous biphasic system involving the relatively mild base Et_3_N, where water was found to be critical in assisting this catalytic system applied to aminations (**Scheme** **11**).^[^ ^61^ ^]^ Using an OAC previously developed for base‐sensitive Suzuki–Miyaura couplings,^[^ ^62^ ^]^ its utility in a 1:4 toluene:water mix was clearly demonstrated. Mechanistic studies indicated that the water was responsible for replacing the halide, affording a cationic Pd(II) species (**Figure** **3**). This is traditionally difficult to do in nonpolar solvents and allowed for a relatively mild intramolecular deprotonation of the formed Pd‐amido species. This system was shown to be amenable toward a variety of base‐sensitive substrates bearing nitriles, esters, and even free carboxylic acids. Additionally, they were able to selectively couple bromides over chlorides based on reaction temperature differences (80 °C vs. 100 °C). Although recycling of the water and/or toluene was not documented, this seems very reasonable given the facility with which these components can be separated and, hence, overall, would significantly decrease the organic waste being produced.

## 3 Sustainable Aminations Using Nontraditional Solvents

Alternative approaches that provide greener solutions to existing methodologies that rely on traditional organic solvents have appeared in the recent literature. Hence, the discussion that follows highlights these contributions in this category that have recently appeared.

### 3.1 Greener, Bio‐Based Solvents

#### 3.1.1 2,2,5,5‐Tetramethyloxalane (TMO)

**Figure 4:**
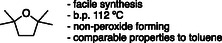
Solvent TMO and the benefits associated with its use.

**Scheme 12:**
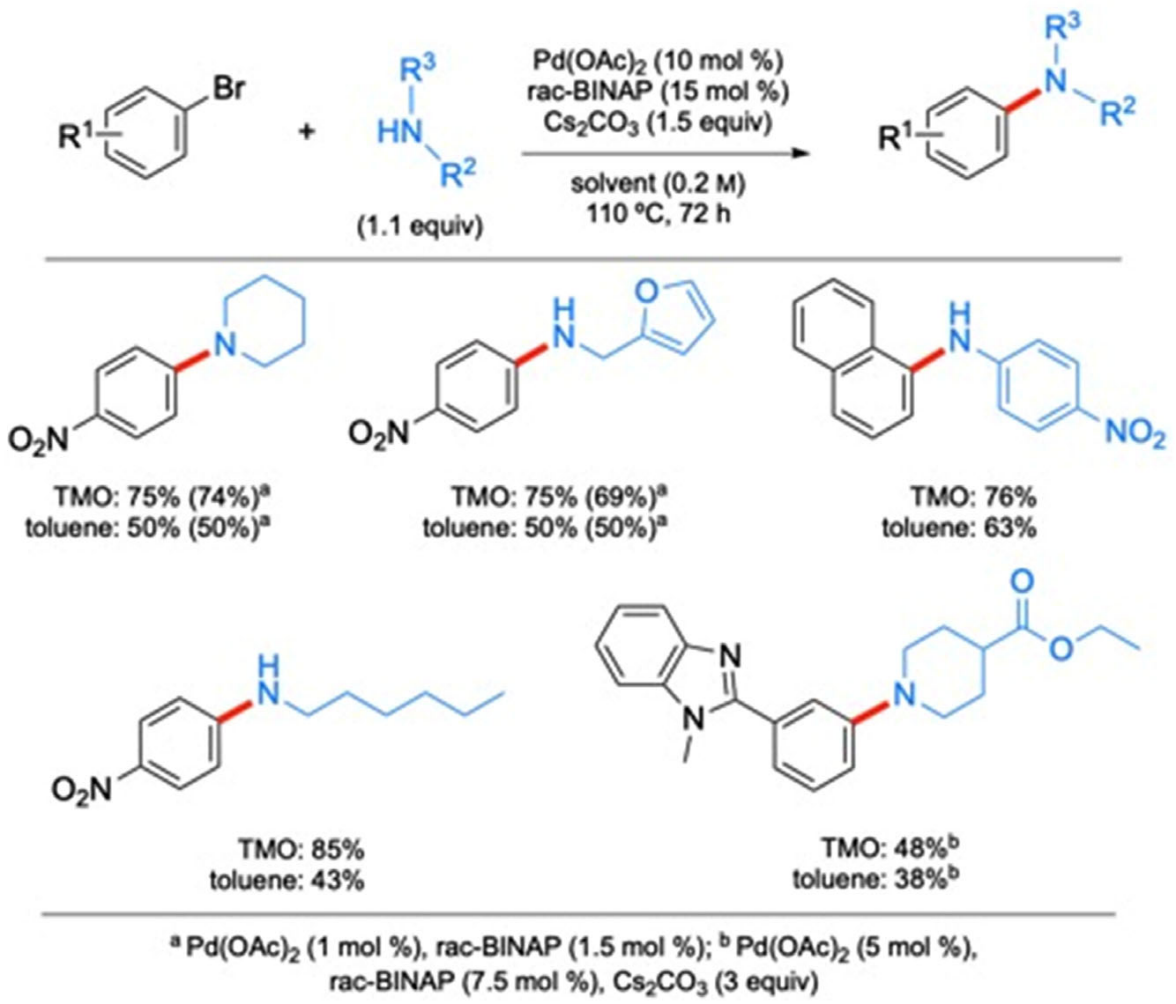
Selected scope for the aminations in TMO compared to toluene. a) Pd(OAc)_2_ (1 mol %), rac‐BINAP (1.5 mol %); b) Pd(OAc)_2_ (5 mol %), rac‐BINAP (7.5 mol %), Cs_2_CO_3_ (3 equiv).

A greener alternative solvent for aminations, introduced by Farmer et al. is 2,2,5,5‐tetramethyloxalane (TMO; **Figure** **4**).^[^ ^63^ ^]^ It is derived from methyl levulinate^[^ ^64^ ^]^ or via catalytic dehydration of 2,5‐dimethyl‐2,5‐hexanediol. With comparable properties to toluene,^[^ ^65^ ^]^ it can be an excellent replacement medium since toluene is considered problematic according to GSK's CHEM21 solvent selection guide.^[^ ^66^ ^]^ Comparisons were made using the commonly employed Pd(OAc)_2_/rac‐BINAP catalyst system and either *t*‐BuONa or Cs_2_CO_3_ as base. The latter, weaker base led to reaction times up to 72 h, while the stronger *t*‐BuONa provided much quicker couplings. Reactions were run using 10 mol % Pd, along with Cs_2_CO_3_ as the base, along with a variety of aryl and aliphatic amines and aromatic bromides. As the examples in **Scheme** **12** show, reactions in TMO gave better results in all cases when Cs_2_CO_3_ was used as base. On the other hand, use of *t*‐BuONa did not produce significant differences in yields between the two solvents. Additionally, they were able to lower the Pd loading to 1 mol % without lowering yields.

#### 3.1.2 Eucalyptol

**Scheme 13:**
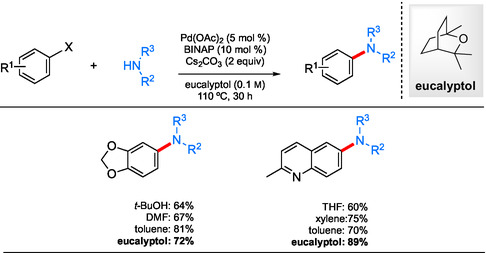
Selected scope for aminations conducted in eucalyptol compared to traditional organic solvents.

Berteina–Raboin et al. investigated eucalyptol as a bio‐based organic solvent^[^ ^67^ ^]^ for use in aminations. It is primarily composed of eucalyptus oil,^[^ ^68^ ^]^ a nontoxic^[^ ^69^ ^]^ naturally occurring substance. Comparisons between couplings of a variety of aryl bromides with amines in both eucalyptol and other organic solvents indicated that this is certainly a viable alternative to several organic solvents (**Scheme** **13**). The recyclability of eucalyptol was also noted adding to its greener nature.

#### 3.1.3 Vegetable Oils

**Scheme 14:**
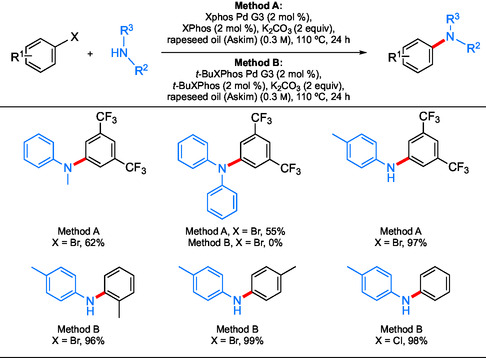
Selected scope for the coupling of aryl halides and amines using vegetable oils.

Chemical waste is not the only significant problem the environment is facing; food waste is also a major concern.^[^ ^70^ ^]^ Gevorgyan and co‐workers described a study using lipids and vegetable oils as safe and sustainable solvents for Pd‐catalyzed aminations.^[^ ^71^ ^]^ They surveyed a variety of oils and found that rapeseed, coconut, and olive oil all gave comparable, quantitative results compared with solvents such as 2‐MeTHF, dioxane, and dimethylformamide (DMF). Moreover, a variety of additives were found to increase the yields of aminations for low‐performing oils, such as those from fish, sunflower, soybean, and avocado. Using high‐resolution mass spectrometry analysis, it was determined that rapeseed oil contains a variety of glycerol, free fatty acids, and mono‐ and di‐glycerides which they suggested act as amphiphiles creating reverse micelles that aid the aminations. These minor impurities were shown to be responsible for the improvements observed by screening the effect of pure additives on low‐performing oils. Using either XPhos‐ or *t*‐BuXPhos‐ligated Pd catalysts, several amination products were formed in rapeseed oil in high yields (**Scheme** **14**). Importantly, recycling and reuse of the oil were possible simply by filtering through Celite followed by drying under reduced pressure.^[^ ^71^ ^]^

### 3.2 Sustainable Catalyst Design

#### 3.2.1 Solvent‐Free Aminations

**Scheme 15:**
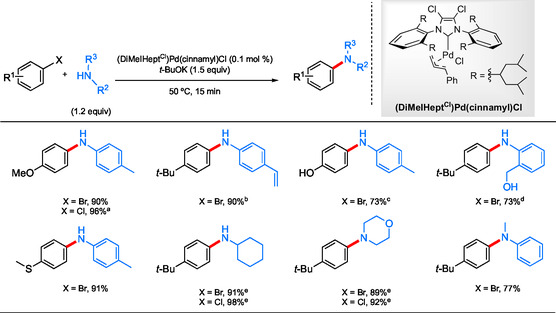
Selected scope using (DiMeIHept^Cl^)Pd(cinnamyl)Cl precatalyst in “melt” reactions. a) 80 °C; b) HMDS (2 mol %), BHT (1 mol %); c) *t*‐BuOK (2.7 equiv), Al(O‐*t*‐Bu)_4_ (1.25 equiv); d) *t*‐BuOK (2.7 equiv), HMDS (1.25 equiv) 1 h; e) 120 °C.

Organ et al. developed a novel ligand that functions in the complete absence of a reaction medium.^[^ ^72^, ^73^ ^]^ Their designer *N*‐heterocyclic carbene (NHC) allowed for a “melt” reaction where the long lipophilic arms of the NHC helped protect the Pd core while also creating an environment where the oxidative addition would proceed smoothly on the basis of London dispersion forces with the aryl halide. This methodology is amenable toward a variety of amine nucleophiles coupling with aryl chlorides and bromides (**Scheme** **15**). Selective coupling of amines over both benzylic and aromatic alcohols was also achieved. With Pd loadings as low as 0.1 mol %, along with typically moderate reaction temperatures (50 °C), these couplings make significant strides in sustainability through the reduced demand for energy, precious metal, and solvents (or lack thereof).^[^ ^4^, ^74^ ^]^ To truly demonstrate the utility of this methodology, several reactions on the gram scale were run, producing comparable yields to those realized on a smaller, academic scale.

#### 3.2.2 Palladium Precatalysts Using Greener Auxiliaries

**Scheme 16:**
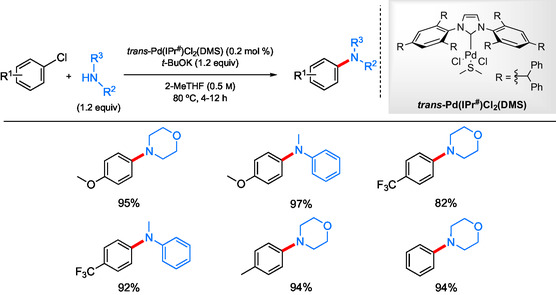
Selected scope for aminations using *trans*‐Pd(IPr^#^)Cl_2_(DMS) as precatalyst.

The use of precatalysts has become quite common, as they are often more stable, easier to handle, and provide more reproducible results. In addition, yields may be higher when activated, leading to freshly generated catalysts containing the corresponding Pd^0^ species.^[^ ^75^ ^]^ Liu et al.^[^ ^76^ ^]^ described a novel approach to palladium catalysts using a safer “throwaway” ligand for Pd–NHC precatalysts similar to the PEPPSI ligand reported by Organ et al.^[^ ^77^, ^78^ ^]^ The use of either dimethylsulfide (DMS) or tetrahydrothiophene (THT) provided safer and easier‐to‐purify, inexpensive ligands compared with PEPPSI‐IPent. Screening a variety of precatalysts represented by *trans*‐[Pd(NHC)Cl_2_(DMS/THT) complexes, Pd(IPr^#^)Cl_2_(DMS) was chosen to demonstrate the utility of these species for use in Pd‐catalyzed aminations (**Scheme** **16**). Notably, the reactions were run in (potentially) recoverable and recyclable 2‐MeTHF as a greener alternative solvent at 80 °C. A variety of 1° and 2° amines, both aliphatic and aromatic, were readily coupled with electron‐rich and ‐poor aryl chlorides. The anilines selected as reaction partners usually contained sterically demanding groups ortho to the amine. Moreover, the use of these DMS‐based “throwaway” ligands adds to the goals of sustainability due to both their atom economy as well as their safety features.

## 4 Aminations via Continuous Flow and Mechanochemical Processes

While continuous flow has emerged as a very useful means of effecting organic synthesis, its use as an option from the perspective of green chemistry remains rather limited. This approach, in general, offers several well‐established advantages, including improved mixing, reduced reaction times, safer handling conditions, and usually, waste reduction.^[^ ^79^ ^]^ Pd‐catalyzed aminations, among many other reaction types, stand to benefit from flow technologies if the principles of green chemistry are fully considered along the way, which is not the case, as high palladium loadings, long reaction times, and usage in organic solvents characterize the norm.^[^ ^26^ ^]^ In considering C—N formations in flow, the insolubility of starting materials, products, and especially inorganic salt byproducts in organic solvents can create major problems due to the clogging of lines, which ultimately leads to reactor failure. Since plug flow is commonly conducted in a coil or packed‐bed reactor(s), it requires the system to flow freely under steady‐state equilibrium conditions without interruption in the form of precipitates.^[^ ^79^, ^80^ ^]^ This has been a particular challenge for conducting Pd‐catalyzed aminations in flow.

Another alternative to using a traditional batch method of synthesis is mechanochemistry. One of the most important principles of green chemistry revolves around the choice of solvent, where “no solvent is the best solvent…”^[^ ^81^ ^]^ Mechanochemistry involves solventless conditions, where mechanical force is the main initiator of the chemical reaction.^[^ ^82^, ^83^ ^]^ Using either shaker or planetary milling methods, spherical balls mechanically grind the reaction mixture in an enclosed system. The key to success of mechanochemical synthesis is often the use of either liquid‐assisted grinding (LAG)^[^ ^84^ ^]^ or auxiliary grinding.^[^ ^85^ ^]^ Either involves an additive used catalytically (e.g., NaCl, Na_2_SO_4_, silica, and trace liquid), which assists the desired transformation, potentially by disrupting intramolecular interactions. As mechanochemistry gains more momentum in the chemical community, there remains a need to demonstrate its sustainable use in parallel reactions^[^ ^86^ ^]^ (multiple reactions run sequentially), as well as applications toward a variety of transition‐metal‐catalyzed processes.^[^ ^87^ ^]^

### 4.1 Aminations in Water via Plug Flow

**Scheme 17:**
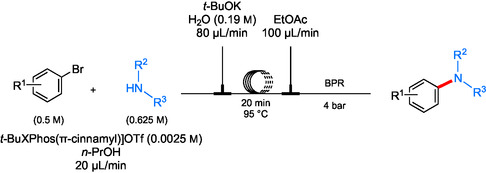
Reactor schematic for the aminations of aryl bromides in continuous flow under aqueous conditions.

**Scheme 18:**
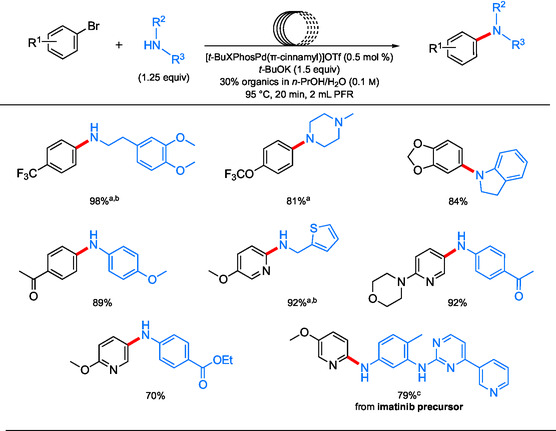
Selected scope for aminations under aqueous conditions in continuous PFR. a) [*t*‐BuXPhosPd(π‐cinnamyl)]OTf (1 mol %); b) *t*‐BuXPhos (3 mol %); c) THFA used a co‐solvent.

There have been several attempts at solving the problem of formation of solids during continuous flow, especially concerning Pd‐catalyzed aminations.^[^ ^88^ ^]^ One setup of a plugged‐flow reactor consists of a VapourTec SF‐10 peristaltic pump for purposes of delivering aqueous *t*‐BuOK, while Harvard Apparatus syringe pumps deliver organics dissolved in *n*‐PrOH into a Teflon PFA (perfluoroalkyl) coil reactor in an oil bath set to 95 °C and a back‐pressure regulator at 4 bar (**Scheme** **17**). The use of water as the main reaction medium allowed for medium recycling following degassing (with argon) overnight and then the addition of *t‐*BuOK as base, to remake the stock solution for a subsequent amination. This aqueous system in flow may help prevent reactor failure that could be caused by the clogging of lines from inorganic salt byproducts. The utilization of both (hetero)aromatic and aliphatic amines (both, 1° and 2°) led to coupled products (i.e., amines) starting with a variety of aryl bromides in relatively high yields (**Scheme** **18**).

### 4.2 Ionic Liquid‐Forming Organic Bases in Plug Flow

**Scheme 19:**
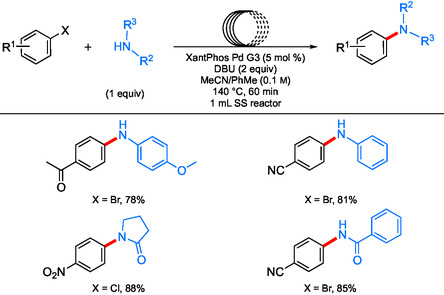
Aminations and amidations in continuous flow using DBU as organic base.

Another option explored by Newman et al.^[^ ^89^ ^]^ showcases DBU as an organic base for Pd‐catalyzed aminations in flow. Using high‐throughput experiments, a variety of DBU salts, DBU·HX, were created that form a low‐melting ionic liquid (e.g., DBU·HCl; mp 66 °C).^[^ ^80^ ^]^ Reactions run at 140 °C, which is well above the melting point at which the ionic liquid forms, and using a mixture of acetonitrile and toluene ensured solubility of materials and, specifically, the protonated base byproducts, which are liquids at these temperatures. With the combination of commercially available catalyst XantPhos Pd G3 and DBU as base, a variety of couplings were demonstrated, including both aminations and amidations (**Scheme** **19**). It is worth noting that the use of this weaker base in conjunction with XantPhos led to couplings of amides.

### 4.3 Biphasic Aminations in Plug Flow

**Scheme 20:**
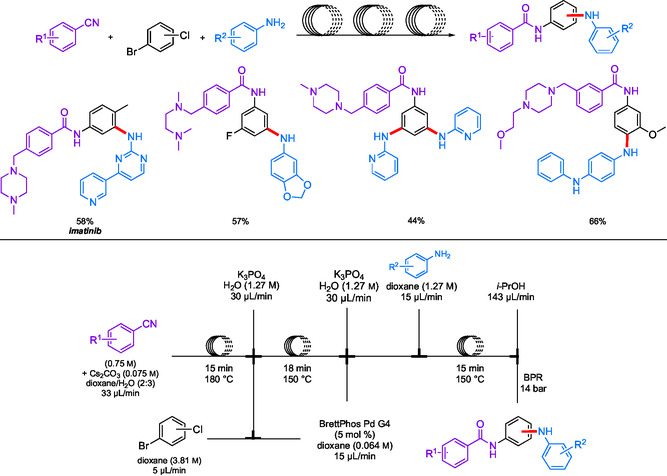
Multicomponent coupling of aryl halides toward imatinib and analogs in modular continuous flow.

**Scheme 21:**
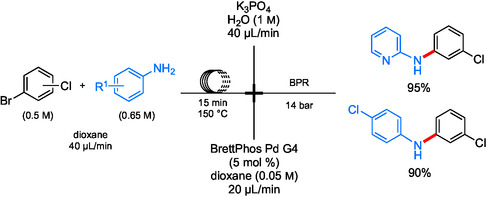
Reactor schematic for the selective aminations of aryl bromides in continuous flow.

Biphasic systems have been designed with water, enabling the dissolution of the inorganic salts formed during the reaction. Modular flow was utilized in a synthesis of imatinib and analogs (**Scheme** **20**) by Jamison et al. involving a biphasic system for both amidations and aminations.^[^ ^90^ ^]^ A mixture of aqueous K_3_PO_4_ and BrettPhos Pd G4, along with starting materials in dioxane, was used giving rise to several products in relatively high yields (**Scheme** **21**). This approach (i.e., an aqueous biphasic system) helped to solubilize the solid phosphate salt byproducts, thereby preventing clogging.

**Scheme 22:**
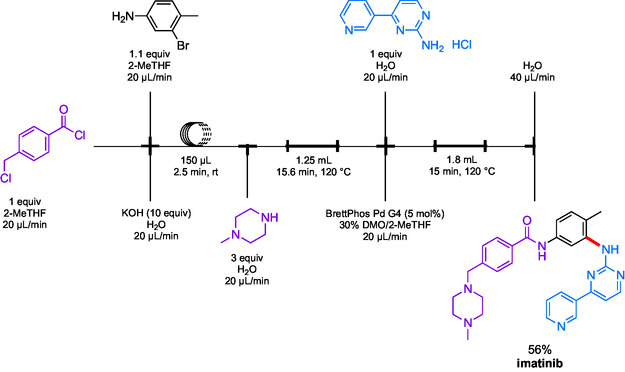
Multicomponent reactions in continuous flow, including an amination en route to imatinib.

In another example, Buchwald et al. used *N,N*‐dimethyloctanamide (DMO) as a cosolvent for a biphasic aqueous/2‐MeTHF system applied to a total synthesis of imatinib (**Scheme** **22**).^[^ ^91^ ^]^ Beginning with an amide coupling using a benzoyl chloride, the product was directed into a second reactor, which led to an S_N_2 with 1‐methylpiperazine. Finally, this crude reaction mixture was delivered into a third reactor containing the required amine (as its HCl salt dissolved in the aqueous phase), which, together with the catalyst dissolved in 2‐MeTHF, afforded the targeted drug in 56% yield over three steps.

### 4.4 Catalyst Recycling

**Scheme 23:**
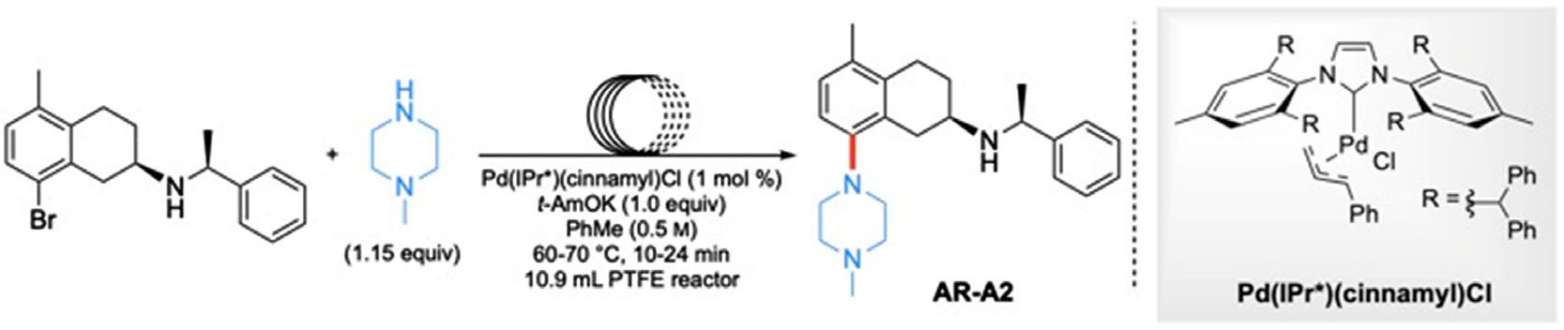
Amination toward the CNS drug AR‐A2 using continuous flow using catalyst recycling.

A route toward the central nervous system (CNS) drug AR‐A2^[^ ^92^, ^93^ ^]^ was reported by Meadows and co‐workers from AstraZeneca, where a recyclable system was developed focused on the catalyst and organic medium (**Scheme** **23**).^[^ ^94^, ^95^, ^96^ ^]^ Their flow system utilized an in‐line acidic workup that was titrated such that the product was selectively protonated, leaving behind the reusable catalyst and any unreacted starting bromide in the organic stream, which was cycled back into the reactor with additional base and starting materials. Their base of choice was *t‐*AmOK, which gets washed away along with byproduct salts.

### 4.5 Aminations via Mechanochemistry

**Scheme 24:**
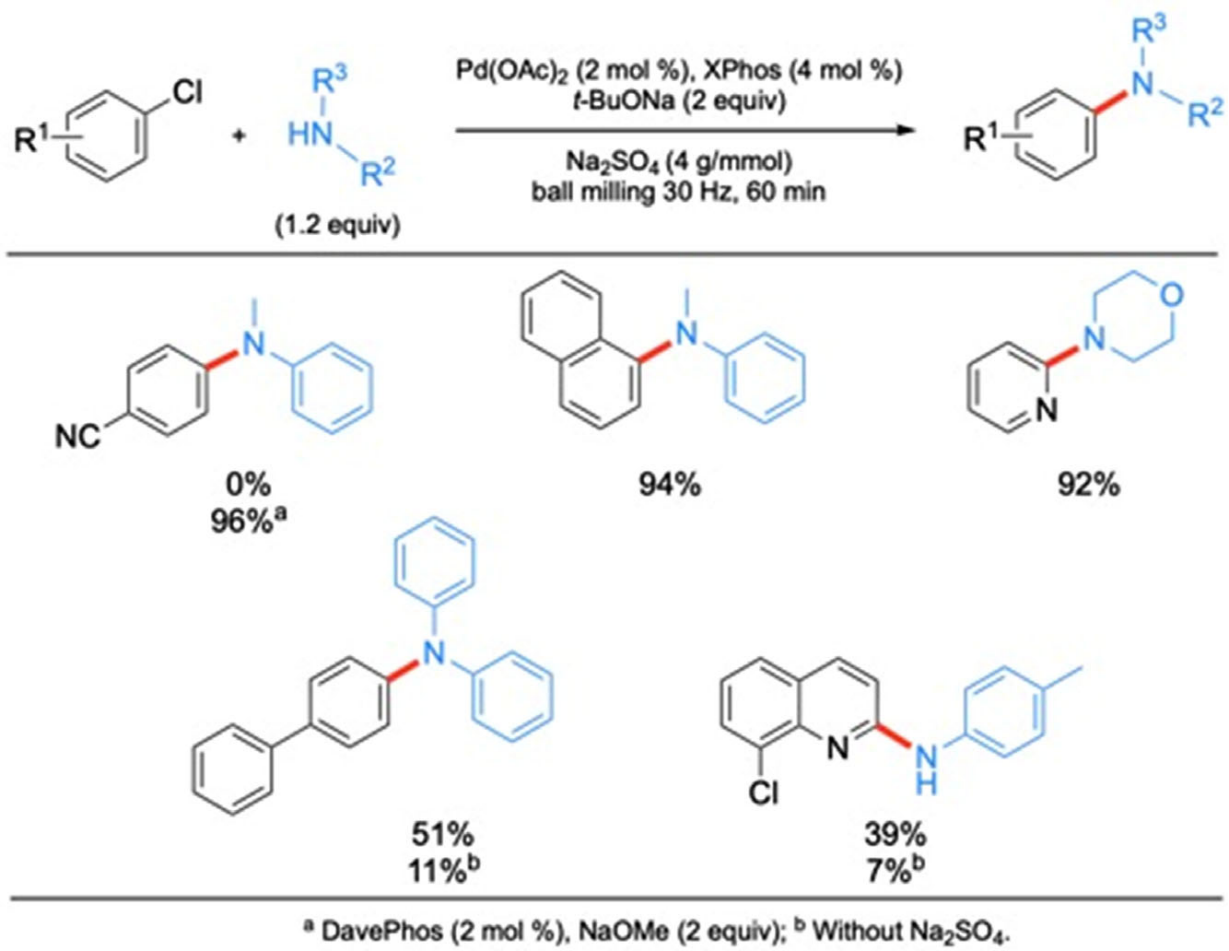
Selected scope for the solvent‐free ball milling mechanochemical aminations using Na_2_SO_4_. a) DavePhos (2 mol %), NaOMe (2 equiv); b) without Na_2_SO_4_.

**Scheme 25:**
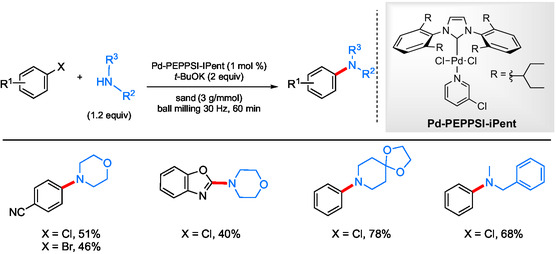
Selected scope for the solvent‐free ball milling mechanochemical aminations using sand.

The first examples of solventless ball milling were reported in 2018 by Su and co‐workers, who showed that aryl chlorides could undergo aminations (**Scheme** **24**).^[^ ^97^ ^]^ The use of solid phase chemistry allowed these reactions to be run under ambient conditions and in the absence of an inert gas atmosphere. After optimization, their system relied on the combination of catalytic Pd(OAc)_2_ and XPhos, with *t*‐BuONa as base, all of which led to a variety of coupling products. The additive Na_2_SO_4_ (4 g mmol^−1^) was found to ensure better grinding. Browne et al. then, in 2019, utilized commercially available Pd‐PEPSSI‐IPent as a precatalyst for related ambient temperature aminations using sand (3 g mmol^−1^) as an additive for ball milling (**Scheme** **25**).^[^ ^98^ ^]^ This system is amenable toward a variety of aryl iodides, bromides, and chlorides, which couple well with 2° alkyl amines. They postulated that the amination occurs so quickly under (solventless) ball milling that catalyst deactivation does not compete with its ability to generate the desired reaction, even under ambient conditions.

**Scheme 26:**
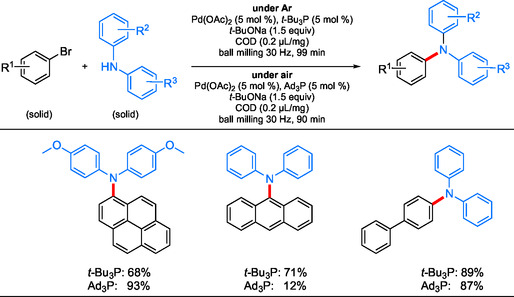
Representative cases of sterically hindered and insoluble couplings conducted using LAG ball milling.

Ito et al. showed in 2019^[^ ^99^ ^]^ and then again in 2020^[^ ^100^ ^]^ that the additive 1,5‐cycloctadiene (COD) was beneficial to conversion for couplings between solid aryl bromides and solid amines (**Scheme** **26**). After screening a variety of additives that could function for purposes of LAG, inclusion of COD increased yields, as did other olefin‐containing over nonolefin‐containing additives. Using transmission electron microscopy imaging, they showed that small added amounts of COD (0.2 μL mg^−1^) led to the generation of Pd NPs of reduced sizes, either accelerating the rate of catalyst (L_n_Pd^0^) formation or preventing aggregation into larger NPs leading to catalyst deactivation. While initial studies utilized Pd(OAc)_2_/*t*‐Bu_3_P (5 mol %), a switch to air stable Ad_3_P allowed for aminations to be run without inert atmosphere conditions, thereby eliminating the potential need for specialized equipment (i.e., Schlenk lines, glove boxes, and so on). Although this work showcases the reduction of organic solvents and inert gas requirements, the reported use of large quantities of metal (5 mol %) remains as a parameter that must be addressed in future studies.

## 5 Sustainable Aminations in Industry

**Scheme 27:**
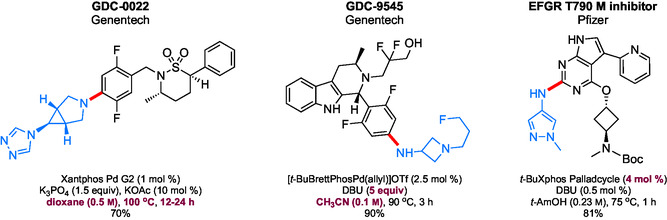
Representative examples of reported, unsustainable APIs that involve Pd‐catalyzed C—N cross couplings.

As industries continue to develop additional products in support of new chemical and biological challenges, as well as in response to a growing worldwide population, the associated costs and waste being generated will also increase. Thus, sustainable practices in industry, due to a variety of factors, will become, over time, an even greater topic of concern. Although improvements have been made, aminations that rely on 1–5 mol % palladium, organic solvents, and high temperatures remain all too common and are not sustainable (**Scheme** **27**).^[^ ^101^, ^102^, ^103^ ^]^

**Scheme 28:**
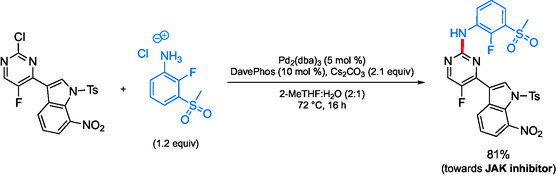
Sustainable amination toward a Janus kinase inhibitor (JAK) inhibitor.

In the pursuit of greener amination conditions, AstraZeneca developed a biphasic system for palladium‐catalyzed C—N formation (**Scheme** **28**).^[^ ^104^ ^]^ This methodology utilizes a 2:1 volume ratio of 2‐MeTHF:water to replace dioxane and DMF as solvents for these couplings, a process which has been shown to be scalable. The reported use of 6–10 mol % palladium loadings, however, is too high from the environmental and cost perspectives.

**Scheme 29:**
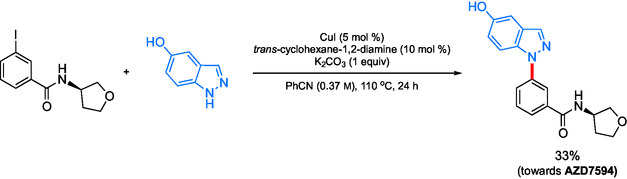
Ligated Cu‐catalyzed amination en route to AZD7594.

AstraZeneca's original multikilo synthesis of AZD7594, an anti‐inflammatory inhalant API candidate, features a C—N cross coupling reaction utilizing copper iodide as the metal catalyst.^[^ ^105^ ^]^ This was pursued on the basis of copper being a safer and environmentally attractive Earth‐abundant metal as compared to palladium (which more recently has been shown using a LCA to be misguided).^[^ ^106^ ^]^ This approach, however, utilizes benzonitrile at elevated temperatures and leads to side products as impurities such that only a 33% yield of the desired product was obtained (**Scheme** **29**).

**Scheme 30:**
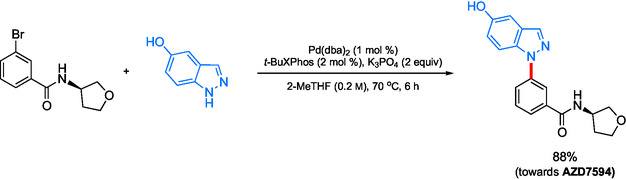
Pd‐catalyzed amination leading to AZD7594.

In recognition of the limitations associated with this copper‐catalyzed approach, a palladium‐catalyzed amination was then pursued. The required C—N bond formation could be achieved using 0.5 mol % Pd in refluxing 2‐MeTHF, affording the target in 88% yield (**Scheme** **30**). However, upon increasing the scale, 1 mol % of Pd was required to prevent incomplete conversion, tantamount to questioning this reaction's reproducibility. Ultimately, this method led to a cleaner reaction, lower metal loading and reaction temperature, and a switch to a greener solvent, thereby reducing the carbon footprint of the process.

**Scheme 31:**
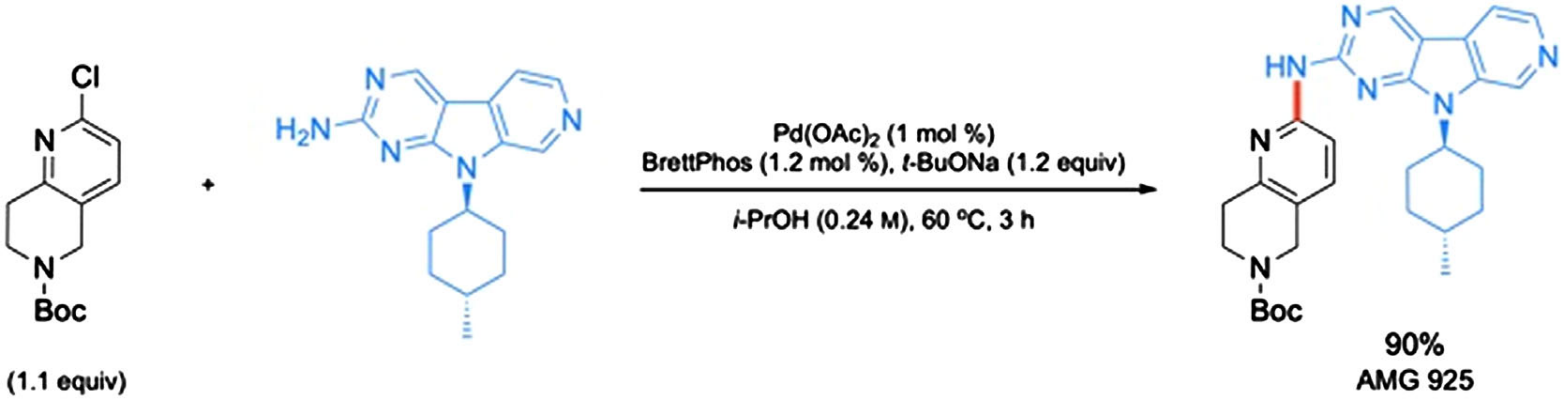
Sustainable amination toward AMG 925.

A multikilo synthesis of Amgen's AMG 925 has also been described, featuring a Pd‐catalyzed amination to form one of the key fragments (**Scheme** **31**).^[^ ^107^ ^]^ This bond formation was initially attempted using metal‐free methods, but none were successful, as <10% conversion was obtained of the desired aminopyrimidine. Utilizing 1 mol % of a ligated source of Pd at 100 °C in *t*‐AmOH, the desired product could be obtained. However, upon further solvent screening, the use of *i*‐PrOH at 60 °C provided similar yields, along with a faster reaction (3 vs. 21 h). The solvent swap was also beneficial for purification, as the product is insoluble in *i*‐PrOH and crashes out of solution upon formation, thereby allowing for a simple filtration to afford the purified product.

## 6 Summary and Outlook

Over the past 40+ years since the initial reports of Pd‐catalyzed aminations between (hetero)aryl halides and amines (see Scheme 1), there has been considerable effort made toward expanding not only the scope of this crucial cross‐coupling, but also the nature of the technology reflecting an awareness of the environmental impact being made by C—N bond constructions. Most traditional methods, nonetheless, still take place in egregious organic solvents (e.g., dioxane, THF, and DME), oftentimes over long reaction times and at elevated temperatures, and use unsustainable levels of precious metal catalysts (typically 1—10 mol % palladium, or more^[^ ^108^ ^]^). Since organic solvents are well known to generate a large percentage of waste being produced, replacements have been the primary focus en route to more sustainable methods of amination. One approach is based on aqueous micellar catalysis, which offers considerable scope, along with recyclability of the aqueous medium. Another method using a biphasic mixture consisting of water and toluene allows for similar outcomes. Other less obvious reaction media, such as TMO, eucalyptol, and vegetable oil, have also been shown to be viable for aminations. The development of catalysts that either allow for neat (i.e., solvent‐free), safe reactions, as well as those containing safer auxiliary ligands in greener solvents leading to C—N bond formation, are also now available for consideration. Given these advances, it becomes a challenge to know which offers the most promise; which is the greenest? and is there one, in particular, that should be highlighted as leading such an important field toward achieving true sustainability? If only it were that simple, a “one‐size‐fits‐all” technology would stand alone among all of these options. But since it is well‐documented that several processes now exist as greener alternatives to those using organic solvents, perhaps the best response is to fall back on the words of Roger Sheldon and co‐workers, who in their monograph dating back to 2007^[^ ^109^ ^]^ taught the world (on page 27 therein) that “The best solvent is no solvent, and if a solvent (diluent) is needed then water is preferred.” Even using these words as a guiding principle in determining sustainable technologies regardless of substrate, conditions, etc., there remains much room for future contributions to this and many other areas of organic synthesis, as green chemistry continues to infiltrate both academic and industrial labs.

## Conflict of Interest

The authors declare no conflict of interest.
